# Association of androgen with gender difference in serum adipocyte fatty acid binding protein levels

**DOI:** 10.1038/srep27762

**Published:** 2016-06-08

**Authors:** Xiang Hu, Xiaojing Ma, Xiaoping Pan, Yuqi Luo, Yiting Xu, Qin Xiong, Yuqian Bao, Weiping Jia

**Affiliations:** 1 Department of Endocrinology and Metabolism, Shanghai Jiao Tong University Affiliated Sixth People’s Hospital, Shanghai Clinical Center for Diabetes, Shanghai Key Clinical Center for Metabolic Disease, Shanghai Diabetes Institute, Shanghai Key Laboratory of Diabetes Mellitus, Shanghai, 200233, China

## Abstract

Clinical investigations have indicated women have higher levels of adipocyte fatty acid binding protein (A-FABP) than men. The present study aimed to identify factors related to gender difference in serum A-FABP levels. A total of 507 participants (194 men, 132 premenopausal women, and 181 postmenopausal women) were enrolled in the present study. Serum A-FABP levels increased in the order from men to premenopausal women to postmenopausal women in both body mass index categories (<25.0 and ≥25.0 kg/m^2^; all *P* < 0.05). Multiple stepwise regression analyses showed that after adjustment for factors related to serum A-FABP levels, the trunk fat mass was an independent and positive factor of serum A-FABP levels. For men, total testosterone was associated independently and inversely with serum A-FABP levels. For pre- and postmenopausal women, bioavailable testosterone and total testosterone were independent and positive factors associated with serum A-FABP levels, respectively. The present study demonstrated that the androgen was correlated with the serum A-FABP levels negatively in men, but positively in women. With these effects on the fat content, especially trunk fat, androgen might contribute to the gender difference in serum A-FABP levels.

Adipocyte fatty acid binding protein (A-FABP), also referred to as fatty acid binding protein 4 and adipocyte protein 2, is one of the most abundant transport proteins in mature adipocytes^1^. Functioning as a positive factor in fatty acid signaling, A-FABP influences the intracellular trafficking of fatty acids and other lipophilic substances by targeting and delivering fatty acid metabolites to the lipid signal transduction pathway directly^1^.

A-FABP is universally recognized as a central player in obesity and obesity-related diseases, such as type 2 diabetes mellitus, metabolic syndrome, and atherosclerosis^2^,^3^,^4^,^5^, due to its effects on lipid and glucose metabolism, insulin resistance, inflammation, reactive oxygen species generation, and endothelial function^6^,^7^,^8^. Notably, most clinical studies examining serum A-FABP levels have reported gender difference, with higher levels observed in women versus men^6^,^7^,^8^. However, the underlying mechanism for this gender difference has not been fully elucidated.

Clinical studies with small sample sizes have suggested that the serum A-FABP levels correlate with concentrations of sex hormones^9^,^10^. Sex hormones play critical roles in the production and distribution of fat, leading to a gender dimorphism in body composition^11^,^12^,^13^,^14^. Accordingly, it is reasonable to hypothesize that sex hormones may have different fat-induced effects on serum A-FABP levels in each gender, and if true, this hypothesis offers an explanation for the gender difference in serum A-FABP levels. Until now, few studies with large sample sizes have explored the relationships between serum A-FABP levels, sex hormones, and fat content and distribution in both genders.

Bioelectrical impedance analysis (BIA), a non-invasive, inexpensive, and simple technique for assessing body composition, is recommended for epidemiological studies because it facilitates the evaluation of a large cohort of individuals within a short period of time^15^. Using BIA to determine body composition, the present study took serum A-FABP levels, sex hormone levels, as well as fat content and distribution into consideration. Overall, we aimed to investigate whether body fat content and distribution can explain the gender difference in serum A-FABP levels. Our previous research demonstrated close associations of circulating A-FABP levels with diabetes and diabetes-induced cardiac dysfunction^16^. Hence, to eliminate the influence of diabetes and cardiovascular diseases on serum A-FABP levels, subjects with diabetes and cardiovascular diseases were excluded from the present study.

## Results

### Clinical characteristics of the study participants

The study population included 507 participants with an age range of 22.42–68.93 years [median, 51.99 (44.57–57.81) years], including 194 men, 132 premenopausal women, and 181 postmenopausal women. In terms of the indexes of body fat, women, whether before or after menopause, had significantly higher values for the total body fat [total fat mass (FM) and total fat percentage (fat%)] and segment body fat (trunk FM, trunk fat%, arm FM, arm fat%, leg FM, and leg fat%) compared to men (all *P* < 0.01). The total fat%, trunk FM, trunk fat%, and arm fat% were higher in postmenopausal women than in premenopausal women (all *P* < 0.05), whereas no difference was observed in the leg FM and fat% (both *P* > 0.05). With respect of sex hormones, compared to men, premenopausal women had lower levels of total testosterone (TT), free testosterone (FT), and bioavailable testosterone (BT) (all *P* < 0.01), but higher levels of estradiol (E_2_) and sex hormone-binding globulin (SHBG) (both *P* < 0.01). As for postmenopausal women, E_2_, TT, FT, and BT were lower than those in men (all *P* < 0.01), and only SHBG (*P* < 0.01) was higher than that in men. The comparison between pre- and postmenopausal women showed that E_2_, TT, and SHBG were much lower in women after menopause (all *P* < 0.05), whereas FT and BT did not differed by the menopause status (both *P* > 0.05). Moreover, compared to men, premenopausal women were younger in age and had a lower body mass index (BMI), waist circumference (W), systolic blood pressure (SBP), diastolic blood pressure (DBP), 2-hour plasma glucose (2hPG), triglyceride (TG), C-reactive protein (CRP), and albumin (Alb) (all *P* < 0.01), but a higher levels of high-density lipoprotein cholesterol (HDL-c; *P* < 0.01). BMI, W, DBP, and serum Alb levels were lower (all *P* < 0.05), while age, glycated hemoglobin (HbA1c), total cholesterol (TC), HDL-c, and low-density lipoprotein cholesterol (LDL-c) (all *P* < 0.01) were higher in postmenopausal women than those in men. Postmenopausal women showed an older age and higher values of W, SBP, 2hPG, HbA1c, fasting serum insulin (FINS), TC, TG, LDL-c, and CRP than premenopausal women (all *P* < 0.05; Table 1).

### Serum A-FABP levels in men, premenopausal women, and postmenopausal women

**Figure 1:**
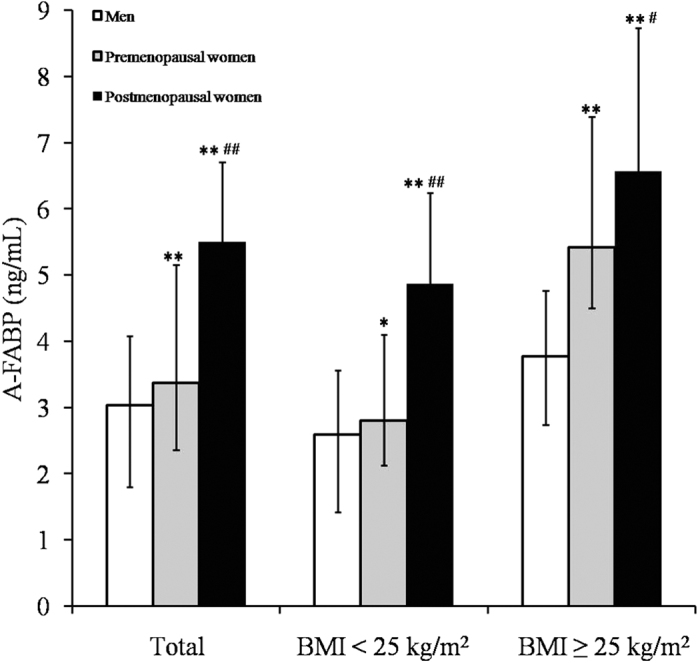
Comparison of serum A-FABP levels between men, premenopausal women, and postmenopausal women. Serum A-FABP levels are expressed as medians with interquartile ranges. ^*^P < 0.05 versus men, ^**^P < 0.01 versus men; ^#^*P* < 0.05 versus premenopausal women, ^##^*P* < 0.01 versus premenopausal women.

Among men, premenopausal women, and postmenopausal women, the serum A-FABP levels were 3.04 (1.80–4.08) ng/mL, 3.37 (2.36–5.16) ng/mL, and 5.50 (3.80–6.71) ng/mL, respectively. Compared to men, both premenopausal women (*P* = 0.004) and postmenopausal women (*P* < 0.001) had higher serum A-FABP levels. Furthermore, serum A-FABP levels appeared to be higher in postmenopausal women than in premenopausal women (*P* < 0.001). Subgroup analysis confirmed the significant differences in serum A-FABP levels between men, premenopausal women, and postmenopausal women in either BMI category (BMI < 25.0 kg/m^2^ and BMI ≥25.0 kg/m^2^; Fig. 1).

### Relationships between different variables and serum A-FABP levels

The correlations of the body fat indexes, sex hormones, as well as other variables with serum A-FABP levels are displayed in Table 2. Partial correlation analyses showed that after adjusted for age, BMI, and W, the indexes of the total and segment body fat were correlated significantly with serum A-FABP levels in both genders (all *P* < 0.001). With regard to sex hormones, TT and SHBG displayed negative associations with the serum A-FABP levels in men (both *P* < 0.001). In women of both groups of menopausal status, however, TT, FT, and BT were positively associated with serum A-FABP levels (all *P* < 0.01), and SHBG was negatively associated with serum A-FABP levels (both *P* < 0.01).

### Multiple stepwise regression analyses of serum A-FABP levels

Defining the serum A-FABP levels as the dependent variable, the present study constructed three regression models to determine the independent factor associated with serum A-FABP levels in men, premenopausal women, and postmenopausal women. Model 1 took into account sex hormones and total FM as independent variables and showed that total FM was an independent and positive factor of serum A-FABP levels in the three groups (all *P* < 0.001). For men, TT was associated independently and inversely with serum A-FABP levels (*P* = 0.001). For pre- and postmenopausal women, BT (*P* = 0.005) and TT (*P* = 0.021) were independent and positive factors associated with serum A-FABP levels, respectively. Model 2 replaced the total FM with the segment FM, including the trunk FM, arm FM, and leg FM. The results showed that the trunk FM stood out as an independent and positive factor of serum A-FABP levels in men, premenopausal women, and postmenopausal women (all *P* < 0.001). The relationships of sex hormones [TT for men (*P* = 0.001) and postmenopausal women (*P* = 0.020), and BT for premenopausal women (*P* = 0.007)] with serum A-FABP levels remained significant. Based on model 2, model 3 further introduced other related factors including age, BMI, W, SBP, DBP, HbA1c, homeostasis model assessment for insulin resistance (HOMA-IR), TG, HDL-c, LDL-c, and CRP into analyses. After adjustment for these related factors, the trunk FM and the sex hormone levels described above were still significantly correlated with serum A-FABP levels (Table 3).

## Discussion

In the present study, serum A-FABP levels increased following the order of men, premenopausal women, and postmenopausal women. These differences in serum A-FABP levels were observed among participants in two BMI categories. Fat content, especially trunk FM, was positively associated with serum A-FABP levels. Notably, the relationship of androgens with serum A-FABP levels was divergent, even opposite, in different genders. The androgen was found to be correlated with serum A-FABP levels negatively in men, but positively in women.

The gender difference of higher circulating A-FABP levels in women compared to men has been observed in populations with diverse characteristics^2^,^3^,^6^,^7^,^8^ and in our previous study^17^. In agreement with these findings, the present study confirmed the serum A-FABP levels increased in the order from men to premenopausal women to postmenopausal women, independent of whether the subjects were overweight/obese.

Circulating A-FABP levels are mainly determined by body fat content and distribution^2^. A close positive association between serum A-FABP levels and the total fat mass has been demonstrated both in lean and overweight/obese individuals^2^,^18^. Additionally, evidence that serum A-FABP levels are positively correlated with not only total body fat content but also central fat accumulation came from an observational longitudinal study of 17 HIV-infected children^19^. Previously, we performed magnetic resonance imaging to obtain a precise measurement of abdominal fat and discovered that serum A-FABP levels are influenced by the visceral fat content in women after menopause^20^. Due to the distinct fat deposition between the trunk and limbs, BIA was applied to assess the segment body composition in the present study, with the purpose of an accurate measurement and a clear distinction of the fat content among the whole body and sub-regions. The results suggested that the serum A-FABP levels exhibited a positive relationship with the FM of every sub-region, and a particularly closer association with the trunk FM.

It had been suggested that in men, androgen is inversely associated with not only the total fat mass, but also the trunk FM and appendicular FM^11^,^14^. In addition, the Japanese researchers observed that serum A-FABP levels significantly increased by 73% after androgen deprivation therapy in patients with prostate cancer, suggestive of a negative association between androgen and serum A-FABP levels^9^. In women, fewer studies have been performed, and the results were not always consistent. It has been generally believed that androgen is positively correlated with total body fat content and involved in the menopause-related visceral fat accumulation^11^,^12^,^13^. Some studies, however, found negative associations between circulating testosterone levels and visceral fat accumulation measured by ultrasound or computed tomography^21^. Another study carried out in women with polycystic ovary syndrome showed that serum A-FABP levels are correlated with hyperandrogenemia^10^. Given the clinical associations described above, we found the gender-specific correlations of androgen with serum A-FABP levels to be similar to the relationships of androgen with the FM. Studies investigating the association of estrogen with the fat content and distribution, nevertheless, have produced inconsistent findings^11^,^12^,^13^,^14^. In addition, few clinical studies have evaluated the correlation between estrogen and serum A-FABP levels. Based on these findings, we proposed in the present study that the fat content and distribution represent a link between androgen and serum A-FABP levels. Our results suggested that androgen contributed to the gender dimorphism of serum A-FABP levels through the gender-specific effects on fat content and distribution.

SHBG is the major plasma transport protein for biologically active androgen and estrogen^22^. Because of the higher affinity of SHBG to androgen, a fluctuation in SHBG levels has a greater impact on the activity and metabolism of androgen. Thus, SHBG has been viewed as an index of androgenicity^23^. BT, the non-SHBG-bound testosterone, reflects only a fraction of the active hormone available in specific tissues. For the first time, the present study found that SHBG was inversely correlated with serum A-FABP levels in both genders. The relationships of FT and BT with serum A-FABP levels were positive in women but absent in men. These findings suggested a potential relationship between the SHBG-related active form of testosterone and the gender difference in serum A-FABP levels.

The mechanism underlying the correlation between androgen and serum A-FABP levels remains to be determined. Heterogeneity by gender with respect to the androgen effects on fat content and distribution has been demonstrated in a variety of basic research studies. Gupta *et al*.^24^ found that dihydrotestosterone suppressed adipogenic differentiation through an androgen receptor-mediated pathway in human mesenchymal stem cells and preadipocytes from men. In addition, lipolysis in preadipocytes increased in the presence of dihydrotestosterone, resulting in a decrease in fat content. In female rhesus macaques, testosterone treatment attenuated lipolysis but enhanced fatty acid uptake and expression of fatty acid synthase, leading to an increase in the fat content^25^. Joyner *et al*.^26^ discovered that more androgen receptors existed in visceral versus subcutaneous preadipocytes in both genders, suggestive of the region-specific effects of androgen. Androgen, therefore, was involved in the regulation of the fat distribution in men and women. A cellular-level study using a proteomics approach identified A-FABP as one of the predominant proteins secreted from adipocytes into the serum^2^. Shi *et al*.^27^ dissected the tissue-specific expression characterization of chicken A-FABP. Western blot analysis only detected expression of A-FABP protein in the abdominal fat tissue. Conversely, obesity-induced hyperinsulinemia inhibited hepatic SHBG synthesis, which further led to a reduction in testicular androgen synthesis. In addition, obesity exerted effects on the peripheral aromatization of androgen^28^. These findings could explain the potential reverse causation to some extent.

In light of the evidence discussed above, we proposed that androgen suppressed fat production in men, whereas it enhanced fat production in women. The effects of androgen were most dominant in the visceral fat. The fat content, especially abdominal fat, was one of the determinants of serum A-FABP levels. Consequently, androgen reduced the serum A-FABP levels by inhibiting fat production in men and raised the serum A-FABP levels by promoting fat production in women, leading to the gender difference in serum A-FABP levels.

There are some limitations in the present study. First, because of the cross-sectional nature of this study, it was difficult to determine the cause and effect sequences of changes in androgen, fat content and distribution, and serum A-FABP levels. Second, the sample size was somewhat small. Prospective studies with a larger sample size are warranted to confirm and generalize the results. Third, the reliance on BIA requires several underlying assumptions and any alterations in body geometry and body water distribution of study population could create discrepancies^29^.

In conclusion, serum A-FABP levels increased following the order of men, premenopausal women, and postmenopausal women progressively. Androgen was correlated with serum A-FABP levels negatively in men, but positively in women. Based on its effects on the fat content, especially the trunk fat, androgen might contribute to the gender difference in serum A-FABP levels.

## Methods

### Subjects

The Shanghai Obesity Study (SHOS), a prospective study, investigated the occurrence and development of metabolic syndrome and related diseases^30^. The present study selected a subgroup of participants from the SHOS cohort. Complete data for the segment body composition was available for the enrolled participants. Each participant underwent an oral glucose tolerance test, and the diagnosis of diabetes was based on the 1999 World Health Organization criteria^31^. Exclusion of participants with diabetes mellitus, cardiovascular disease, liver or renal dysfunction, hyperthyroidism or hypothyroidism, pregnancy, acute infection, psychiatric disease, tumors, and current replacement therapy with systemic corticosteroids, thyroxine, or sex hormones resulted in a final sample size of 507.

The study was conducted in accordance with the Declaration of Helsinki and approved by the Ethics Committee of Shanghai Jiao Tong University Affiliated Sixth People’s Hospital. All of the subjects provided written informed consent prior to their participation in the study.

### Measurements of body fat composition and distribution

An automatic bioelectrical impedance analyzer (TBF-418B; Tanita Corp., Tokyo, Japan) was used to measure the total body FM and fat%, as well as the segment body composition including the FM and fat% of the trunk, arms, and legs. Before the measurement, information about age, gender, and height of the subjects was input into the analyzer. Each participant had to step barefoot into a stand-alone unit and take grips in both hands (alongside the body) during the impedance measurement. Values of segmental FM and fat% were obtained at the end of the analysis.

### Anthropometric and biochemical assessments

The body weight, height, W, and resting blood pressure were measured following standard procedures^6^. BMI was calculated as follows: BMI = weight (kg)/height^2^ (m^2^).

Fasting plasma glucose (FPG), HbA1c, FINS, TC, TG, LDL-c, HDL-c, and CRP levels were measured in morning fasting serum samples. Approximately 2 hours after breakfast, 2hPG levels were determined. Standard laboratory measurements were performed, and HOMA-IR was calculated as described previously^6^.

The serum Alb level was measured by the bromocresol green method on a Hitachi 7600-120 auto-analyzer (Hitachi Inc., Tokyo, Japan). The serum concentrations of E_2_, TT, and SHBG were detected on Abbott Architect i2000SR analyzer using chemiluminescent microparticle immunoassays (kits from Abbott GmbH & Co. KG, Wiesbaden, Germany). The FT and BT were calculated from the TT, SHBG, and Alb values as follows: FT = ([T] − N × [FT])/(K_t_ {[SHBG] − [T] + N[FT]}); BT = N × [FT]; where K_t_ = 1 × 10^9^L/mol, N = K_a_ × C_a_ + 1; K_a_ = 3.6 × 10^4^ L/mol and C_a_ is the albumin concentration^32^. Serum A-FABP levels were measured by a sandwich enzyme-linked immunosorbent assay (Antibody and Immunoassay Services, The University of Hong Kong, Hong Kong) with intra- and inter-assay coefficients of variation of 6.6% and 8.7%, respectively.

### Definition

Overweight/obese was defined as a BMI of ≥25.0 kg/m^2^ based on the 1998 World Health Organization criteria^33^. Menopause was defined as at least 12 consecutive months of amenorrhea without other medical causes^34^.

### Statistical analysis

All statistical analyses were carried out using the SPSS 16.0 statistical software package (SPSS Inc., Chicago, IL, USA). The one-sample Kolmogorov-Smirnov test was performed to determine normality of the data distribution. Data are presented as mean ± standard deviation or median with interquartile range in the case of normal or skewed distributions, respectively. Comparisons between the two groups were carried out using the unpaired Student’s t-test (normal distribution) or the Mann-Whitney U-test (skewed distribution) for continuous data, and the Chi-squared test for categorical variables. Partial correlation analyses were conducted to assess the relationships of serum A-FABP levels with sex hormones levels, indexes of body composition, and other metabolic parameters. Multiple stepwise regression analyses were used to identify independent factors affecting serum A-FABP levels. The threshold of statistical significance was set at 0.05 for two tailed *P*-values.

## Additional Information

**How to cite this article**: Hu, X. *et al*. Association of androgen with gender difference in serum adipocyte fatty acid binding protein levels. *Sci. Rep*. **6**, 27762; doi: 10.1038/srep27762 (2016).

## Figures and Tables

**Table 1:** Characteristics of the study participants.

Variable		Men	Premenopausal women	Postmenopausal women
n		194	132	181
Age (years)		51.48 (43.49–58.89)	44.37 (37.98–48.80)^**^	56.70 (53.68–59.85)^**##^
BMI (kg/m^2^)		23.96 ± 2.90	22.89 ± 3.05^**^	23.39 ± 2.65^*^
W (cm)		85.35 ± 8.69	77.32 ± 8.68^**^	80.64 ± 8.18^**##^
Body composition				
Total	FM (kg)	14.53 ± 5.27	18.33 ± 5.99^**^	19.59 ± 5.21^**^
	fat%	19.97 ± 5.02	30.55 ± 5.93^**^	32.48 ± 4.92^**##^
Trunk	FM (kg)	8.04 ± 3.23	9.70 ± 3.70^**^	10.71 ± 3.25^**#^
	fat%	20.73 ± 6.27	29.00 ± 7.22^**^	31.82 ± 5.85^**##^
Arm	FM (kg)	1.10 (0.80–1.40)	1.40 (1.00–1.98)^**^	1.60 (1.20–2.00)^**^
	fat%	15.25 (13.35–17.96)	27.29 (23.19–33.19)^**^	30.13 (26.23–33.04)^**#^
Leg	FM (kg)	5.38 ± 1.76	7.07 ± 1.73^**^	7.26 ± 1.54^**^
	fat%	20.50 (17.97–22.51)	33.00 (30.26–36.60)^**^	34.50 (32.12–36.53)^**^
SBP (mmHg)		120.00 (112.00–130.00)	110.00 (104.00–121.17)^**^	120.00 (110.00–130.00)^##^
DBP (mmHg)		80.00 (74.50–89.33)	78.67 (70.00–80.67)^**^	79.33 (70.00–85.33)^**^
FPG (mmol/L)		5.21 (4.88–5.53)	5.23 (4.89–5.60)	5.17 (4.88–5.47)
2hPG (mmol/L)		6.84 ± 1.81	6.17 ± 1.57^**^	6.90 ± 1.61^##^
HbA1c (%)		5.5 (5.3–5.7)	5.5 (5.3–5.7)	5.7 (5.5–5.9)^**##^
FINS (mU/L)		6.22 (4.30–9.09)	5.87 (4.32–8.17)	6.69 (4.94–9.60)^#^
HOMA-IR		1.44 (0.93–2.29)	1.42 (0.94–1.96)	1.54 (1.10–2.32)
TC (mmol/L)		4.91 ± 0.85	4.80 ± 0.90	5.65 ± 0.96^**##^
TG (mmol/L)		1.42 (0.90–1.95)	0.91 (0.65–1.33)^**^	1.30 (0.94–1.75)^##^
HDL-c (mmol/L)		1.32 ± 0.30	1.57 ± 0.31^**^	1.57 ± 0.32^**^
LDL-c (mmol/L)		2.96 ± 0.79	2.79 ± 0.77	3.40 ± 0.78^**##^
CRP (mg/L)		0.61 (0.33–1.21)	0.44 (0.16–0.87)^**^	0.69 (0.37–1.52)^##^
Alb (g/L)		48.00 (47.00–50.00)	47.00 (46.00–49.00)^**^	48.00 (46.00–49.00)^**^
A-FABP (ng/mL)		3.04 (1.80–4.08)	3.37 (2.36–5.16)^**^	5.50 (3.80–6.71)^**##^
E_2_ (pmol/L)		88.08 (66.06–113.77)	264.24 (128.45–450.49)^**^	18.35 (18.35–36.70)^**##^
TT (nmol/L)		19.83 (16.86–24.58)	2.03 (1.56–2.56)^**^	1.67 (1.28–2.05)^**##^
FT(nmol/L)		0.37 (0.31–0.44)	0.02 (0.02–0.03)^**^	0.02 (0.01–0.03)^**^
BT(nmol/L)		9.93 (8.19–11.34)	0.63 (0.40–0.90)^**^	0.55 (0.38–0.77)^**^
SHBG (nmol/L)		35.45 (27.73–50.93)	58.00 (43.03–84.35)^**^	52.60 (35.95–72.90)^**#^

Abbreviation: BMI: body mass index; W: waist circumference; FM: fat mass; SBP: systolic blood pressure; DBP: diastolic blood pressure; FPG: fasting plasma glucose; 2hPG: 2-hour plasma glucose; HbA1c: glycated hemoglobin A1c; FINS: fasting serum insulin; HOMA-IR: Homeostasis model assessment-insulin resistance; TC: total cholesterol; TG: triglyceride; HDL-c: high density lipoprotein cholesterol; LDL-c: low density lipoprotein cholesterol; CRP: C-reactive protein; Alb: albumin; A-FABP: adipocyte fatty acid binding protein; E_2_: Estradiol; TT: total testosterone; FT: free testosterone; BT: bioavailable testosterone; SHBG: sex hormone-binding globulin.

Data are expressed as means ± SD or median (interquartile range).

^*^P < 0.05 versus men; ^**^P < 0.01 versus men; ^#^*P* < 0.05 versus premenopausal women; ^##^*P* < 0.01 versus premenopausal women.

**Table 2:** Partial correlation analyses of serum A-FABP levels.

		Men		Premenopausal women		Postmenopausal women	
Adjusted for age		*r*	*P*	*r*	*P*	*r*	*P*
BMI		0.539	<0.001	0.551	<0.001	0.494	<0.001
W		0.550	<0.001	0.533	<0.001	0.367	<0.001
Body composition							
Total	FM	0.576	<0.001	0.587	<0.001	0.506	<0.001
	fat%	0.556	<0.001	0.581	<0.001	0.481	<0.001
Trunk	FM	0.567	<0.001	0.602	<0.001	0.507	<0.001
	fat%	0.549	<0.001	0.574	<0.001	0.490	<0.001
Arm	FM	0.544	<0.001	0.552	<0.001	0.477	<0.001
	fat%	0.544	<0.001	0.571	<0.001	0.481	<0.001
Leg	FM	0.552	<0.001	0.526	<0.001	0.463	<0.001
	fat%	0.485	<0.001	0.575	<0.001	0.443	<0.001
SBP		0.180	0.012	−0.018	0.840	0.171	0.022
DBP		0.246	0.001	0.015	0.866	0.110	0.142
FPG		0.249	<0.001	0.248	0.004	0.134	0.073
2hPG		0.230	0.001	0.030	0.734	0.188	0.011
HbA1c		0.211	0.003	0.280	0.001	0.108	0.148
HOMA-IR		0.359	<0.001	0.308	<0.001	0.353	<0.001
TC		0.115	0.111	0.164	0.061	0.060	0.425
TG		0.430	<0.001	0.329	<0.001	0.373	<0.001
HDL-c		−0.467	<0.001	−0.272	0.002	−0.248	0.001
LDL-c		0.094	0.192	0.144	0.100	0.026	0.725
CRP		0.251	<0.001	0.243	0.005	0.176	0.018
E_2_		−0.016	0.828	−0.063	0.475	0.055	0.466
TT		−0.413	<0.001	0.338	<0.001	0.250	0.001
FT		−0.120	0.096	0.451	<0.001	0.301	<0.001
BT		−0.102	0.160	0.442	<0.001	0.302	<0.001
SHBG		−0.429	<0.001	−0.454	<0.001	−0.233	0.002

Abbreviation: BMI: body mass index; W: waist circumference; FM: fat mass; SBP: systolic blood pressure; DBP: diastolic blood pressure; FPG: fasting plasma glucose; 2hPG: 2-hour plasma glucose; HbA1c: glycated hemoglobin A1c; FINS: fasting serum insulin; HOMA-IR: Homeostasis model assessment-insulin resistance; TC: total cholesterol; TG: triglyceride; HDL-c: high density lipoprotein cholesterol; LDL-c: low density lipoprotein cholesterol; CRP: C-reactive protein; A-FABP: adipocyte fatty acid binding protein; E_2_: Estradiol; TT: total testosterone; FT: free testosterone; BT: bioavailable testosterone; SHBG: sex hormone-binding globulin.

**Table 3:** Multivariate regression analyses on serum A-FABP levels.

Variable	Men				Variable	Premenopausal women				Variable	Postmenopausal women			
	*β*	SE	Standardized *β*	*P*		*β*	SE	Standardized *β*	*P*		*β*	SE	Standardized *β*	*P*
Model 1														
TT	−0.066	0.019	−0.218	0.001	BT	0.895	0.313	0.204	0.005	TT	0.633	0.272	0.152	0.021
Total FM	0.165	0.021	0.489	<0.001	Total FM	0.186	0.024	0.550	<0.001	Total FM	0.224	0.031	0.470	<0.001
Model 2														
TT	−0.067	0.019	−0.222	0.001	BT	0.851	0.311	0.194	0.007	TT	0.636	0.271	0.153	0.020
Trunk FM	0.262	0.035	0.478	<0.001	Trunk FM	0.308	0.039	0.565	<0.001	Trunk FM	0.359	0.050	0.470	<0.001
Model 3														
TT	−0.055	0.018	−0.183	0.002	BT	0.851	0.311	0.194	0.007	TT	0.524	0.265	0.126	0.049
Trunk FM	0.194	0.036	0.354	<0.001	Trunk FM	0.308	0.039	0.565	<0.001	Trunk FM	0.311	0.050	0.406	<0.001
HOMA-IR	0.219	0.088	0.150	0.014						TG	0.743	0.207	0.234	<0.001
TG	0.321	0.060	0.298	<0.001										

Independent variables originally included:

Model 1: E_2_, TT, BT, SHBG, total FM;

Model 2: E_2_, TT, BT, SHBG, trunk FM, arm FM, leg FM;

Model 3: E_2_, TT, BT, SHBG, trunk FM, arm FM, leg FM, age, BMI, W, SBP, DBP, HbA1c, HOMA-IR, TG, HDL-c, LDL-c, CRP.
